# Needs-based planning for the oral health workforce - development and application of a simulation model

**DOI:** 10.1186/s12960-019-0394-0

**Published:** 2019-07-15

**Authors:** Susan Ahern, Noel Woods, Olivier Kalmus, Stephen Birch, Stefan Listl

**Affiliations:** 1 Oral Health Services Research Centre, Cork University Dental School & Hospital, University College Cork, Cork, Ireland; 2 Centre for Policy Studies, Cork University Business School, University College Cork, Cork, Ireland; 3 Section for Translational Health Economics, Department of Conservative Dentistry, Heidelberg University, Heidelberg, Germany; 4 Centre for the Business and Economics of Health, The University of Queensland, Brisbane, Australia; 5 Department of Dentistry - Quality and Safety of Oral Healthcare, Radboudumc (RIHS), Radboud University, Nijmegen, The Netherlands

**Keywords:** Oral health, Workforce planning, Needs-based, Provider supply, Provider requirement

## Abstract

**Background:**

The World Health Organization’s global strategy on human resources for health includes an objective to align investment in human resources for health with the current and future needs of the population. Although oral health is a key indicator of overall health and wellbeing, and oral diseases are the most common noncommunicable diseases affecting half the world’s population, oral health workforce planning efforts have been limited to simplistic target dentist-population or constant services-population ratios which do not account for levels of and changes in population need. Against this backdrop, our aim was to develop and operationalise an oral health needs-based workforce planning simulation tool.

**Methods:**

Using a conceptual framework put forward in the literature, we aimed to build the model in Microsoft Excel and apply it in a hypothetical context to demonstrate its operability. The model incorporates a provider supply component and a provider requirement component, enabling a comparison of the current and future supply of and requirement for oral health workers. Publicly available data, including the Special Eurobarometer 330 Oral Health Survey, were used to populate the model. Assumptions were made where data were not publicly available and key assumptions were tested in scenario analyses.

**Results:**

We have systematically developed a needs-based workforce planning model for the oral health workforce and applied the model in a hypothetical context over a 30-year time span. In the 2017 baseline scenario, the model produced a full-time equivalent (FTE) provider requirement figure of 899 dentists compared with an FTE provider supply figure of 1985. In the scenario analyses, the FTE provider requirement figure ranged from 1123 to 1629 illustrating the extent of the impact of changing parameter values.

**Conclusions:**

In response to policy makers’ recognition of the pressing need to better plan human resources for health and the scarcity of work in this area for dentistry, we have demonstrated the feasibility of producing a workable, practical and useful needs-based workforce planning simulation tool for the oral health workforce. In doing so, we have highlighted the challenges faced in accessing timely and relevant data needed to populate such models and ensure the reliability of model outputs.

## Background

Successful health workforce planning is critical to the sustainability of a healthcare system as it encompasses the delivery of the right care, in the right place, at the right time, by the right number of people, to those most in need [1]. Although health workforce planning dates back to the 1960s, over the past 15 years, there has been a growing body of published health workforce planning literature, broadly covering demand-based, supply-based and more recently a limited number of needs-based planning approaches mainly for physicians [2–5], general practitioners [6, 7] and nurses [3, 8–10]. However, while many health system policy makers recognise the need to better plan human resources, most countries across the globe have struggled to successfully develop and implement health workforce planning models [11, 12]. Of those countries that do engage in model-based workforce planning, the majority have adopted supply-based approaches which do not account for the changing health needs of populations [13, 14]. Additionally, the process itself has many challenges, not least the lack of reliable data [13] and no example of ‘best practice’ has been identified to date [15].

A 2013 review of 26 health workforce planning projection models developed in 18 OECD countries included just 1 dentist model [16]. Although oral health is a key indicator of overall health, wellbeing and quality of life and despite the fact that oral diseases are the most common noncommunicable diseases affecting half of the world’s population [17], planning for dental workforces does not appear to be a priority for policy makers. Traditionally, workforce planning in dentistry has rarely extended beyond a simplistic target dentist-population ratio, a widely used measure for transforming demographic projections into required numbers of dentists. While the dentist-population ratio continues to be used in the workforce planning narrative and as a measure of comparing workforce supply between different countries, increasingly it is regarded as a crude measure. It fails to consider many important factors, not least the level of oral health need which differs between countries, between regions within countries and changes over time [18], and the changing composition of services, how they are delivered and by whom they are delivered [9]. Some work has been conducted in the United Kingdom modelling future dental workforce skill mix and its cost-effectiveness [19–21] and forecasting and comparing the supply of and demand (driven by changes in the projected size and composition of the population only) for National Health Service General Dental Practitioners in Scotland [22]. However, to the best of our knowledge, no comprehensive oral health needs-based population workforce planning simulation model has been put forward to date.

Our aim was to develop a practical oral health needs-based workforce planning simulation tool in Microsoft Excel [23] and apply it in a hypothetical scenario, using publicly available data. In doing so, we also highlight the challenges faced in sourcing ongoing and timely data needed to populate the model and produce robust output, without which the needs-based workforce planning process becomes a theoretical exercise.

## Method

**Fig. 1:**
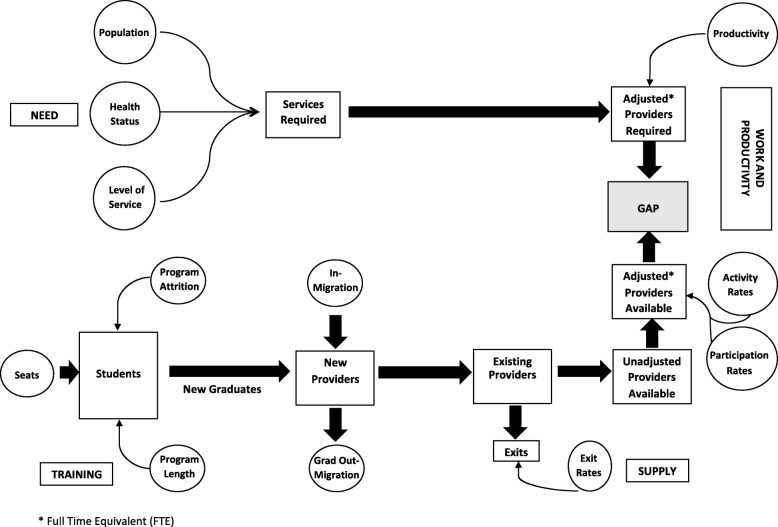
Graphical representation of the needs-based framework [9]

This study is part of the ADVOCATE project (Added Value for Oral Care), funded by the European Commission’s Horizon 2020 programme [24], with six participating countries—Denmark, Germany, Hungary, the United Kingdom, Ireland and the Netherlands. One of the ambitions of ADVOCATE is to develop a needs-based oral health workforce planning model aiming to ensure the provision of the most economical combination of workforce skills needed for the effective, efficient and safe provision of oral health services that can be provided within available resources for both the long and short terms. Using the needs-based workforce planning conceptual framework developed in previous work by one of the current authors and colleagues [14] and provided in Fig. 1 below, we have developed an analytical framework in Microsoft Excel by building a series of linked spreadsheets illustrating how a useful and workable oral health needs-based workforce planning tool can be produced. Although we have applied the model in a hypothetical context, incorporating many data assumptions, in order to present a realistic setting for the application of the model, we have used publicly available Irish data where possible. The model consists of two components: provider supply and provider requirement, both of which are described in detail below.

### Provider supply

The provider supply module is broken down into three sub components: (i) existing stock, (ii) flow and (iii) newly trained. Existing stock is the current supply of licenced practitioners. The flow of practitioners incorporates both inflow and outflow. Inflow includes new registrations but not those who are newly qualified in the country in question. Depending on available data, inflow can be broken down further to provide finer detail on the nature of inflows. These categories may include those trained elsewhere and entering a new country to practice, those who trained abroad and are now returning to practice in their home country and those returning to work following a career break or period of absence. Similarly, outflow of practitioners can be broken down and distinguished further by identifying those emigrating to practice in another country, those taking a career break or a period of absence and those who are retiring and deaths in service. Assumptions regarding inflows and outflows in future years can be based on these figures but also adjusted based on available data. The estimated number of newly trained practitioners available for work in a particular country is calculated as follows: the number of undergraduate places on offer is firstly adjusted for non-progression to year 2 of the course and attrition thereafter. The number of graduates is then adjusted to account for the percentage entering employment in that country. This produces a full-time equivalent (FTE) number of graduates available for work.

Using the stock of practitioners at the end of last year as a starting point for the current year, the number is adjusted for inflow, outflow and new graduates available for work as described above. This produces an estimate of the supply of practitioners practising at the end of the current year. This becomes the starting stock figure at the beginning of the next year, and the process of calculating inflows and outflows continues for each year thereafter of the planning period, producing a figure for stock at the end of each year and the start of the next year.

Before reporting provider supply, the stock figures must be adjusted to account for participation and level of activity. Practitioner registers may include those who may not be actively practicing, for example those in full-time academic positions. To account for this, the figure for provider supply is adjusted using a ‘participation rate’. Furthermore, it is recognised that not all dental practitioners are working full-time hours [25–27]. It is therefore important that a workforce planning model can account for the changing profile and working patterns of those delivering oral healthcare services [28]. Our model accounts for part-time workers by adjusting workforce supply using an ‘activity rate’. This produces a final provider supply figure reported as a FTE number of practising providers.

### Provider requirement

Similar to the provider supply module, the provider requirement module is broken down into three subcomponents: (i) demography, (ii) health status and (iii) service. To illustrate the scope of a needs-based workforce planning model for the oral health workforce using publicly available data, and in the context of a model that could be replicated across multiple European countries, we chose to use the Special Eurobarometer 330 Oral Health Survey dataset [29] containing relevant demography, oral health status and oral health service data. The Special Eurobarometer 330, conducted in October 2009, is part of wave 72.3 of the Eurobarometer covering the population of the respective nationalities of the European Union Member States, resident in each of the Member States and aged 15 years and over. The survey was intended to contribute to meeting one of the main objectives of the European Global Oral Health Indicators Development, namely the description of certain oral health indicators at the European level [30]. The variables detailed in Table 1 below are available within the dataset and have been used in our modelling work.

**Table 1:**
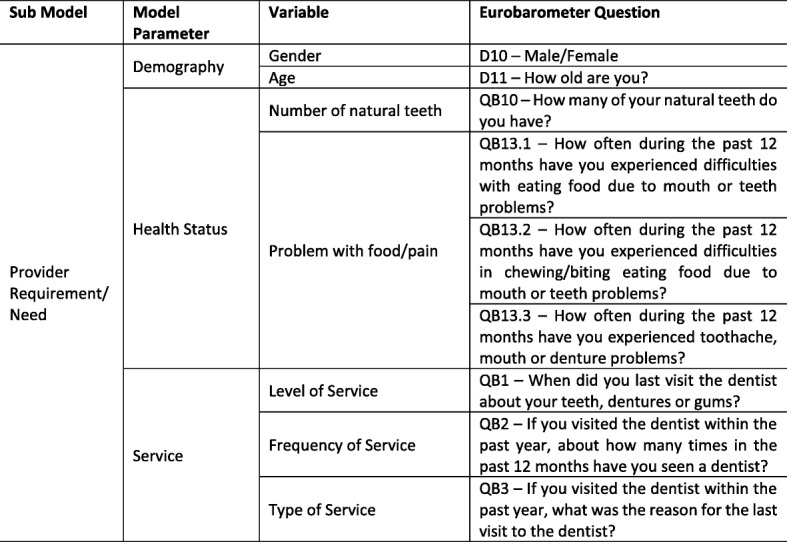
Provider requirement/need variables available from Eurobarometer 330 Oral Health Survey dataset [29]

In calculating provider requirements, the model estimates the number of FTE practitioners required to meet the needs of the adult (15 years and over) population. To do this, we created four gender-specific age cohorts of males and females; 15–44 years, 45–64 years, 65–74 years and 75 years and above. Using sample service data indicating if someone had visited the dentist in the previous 12 months and ‘frequency of service’ in the past 12 months, we were able to establish the total number of visits by gender, age cohort and health status (number of natural teeth and whether someone has a problem with food/pain or not). The total number of visits was then broken down by ‘type of service’, that is, either a check-up/exam/cleaning, routine treatment or emergency treatment. By applying a time component to each of the three ‘types of service’ and multiplying this by the total number of visits, we calculated the total service requirement in minutes by gender, age cohort and health status. The total provider requirement in minutes was converted to an equivalent number of FTE practitioners by dividing the figure by the total minutes worked by a practitioner in a calendar year.

### Application of the model

To operationalise the model and demonstrate its feasibility, we applied the model in a hypothetical context, focusing on the needs of a patient population of adults aged 15 years and over only. We used Irish data (Republic of Ireland), where publicly available, to populate the model but otherwise our inputs have required a number of data assumptions. The time horizon for the workforce planning period is from 2017, for which we have actual population statistics, to 2050.

To calculate dentist supply, we started with the Register of Dentists in Ireland [31] maintained by The Irish Dental Council, under the Dentists Act 1985. The register is published annually and as required and can be used to establish an approximate number for the current stock of dentists. It is noted that although dentists may be registered, they may not be practising as private dentists or practising at all. Inflows were been broken down into three categories; incoming dentists who did not train in Ireland but are newly registered to practice in Ireland, incoming dentists who did train in Ireland are newly registered and are now returning to practice in Ireland and those returning to work following a period of absence, for example a career break. The Register of dentists in Ireland provides detail of ‘Date Registered’, ‘Year Qualified’ and ‘Primary Recognisable Qualification’ of all registered dentists [31]. This enabled us to approximate the number of inflows for the first two categories above in the current year. Assumptions have been made on the numbers returning to the workforce after a period of absence. Assumptions regarding inflows in future years are also based on these figures. With regard to outflow of dentists, a comparison of the register from 2017 to 2018 enabled us to approximate the numbers in each of the outflow subcategories for the current year, that is, dentists leaving the country, those taking a career break or a period of absence and those who are retiring. Assumptions regarding outflows in future years are also based on these figures. The estimated number of newly trained dentists available for work in Ireland was calculated as follows: the number of undergraduate places on offer in the two dental schools in Ireland was firstly adjusted for non-progression to year 2 of the course and attrition thereafter [32]. The number of graduating dentists was then adjusted to account for the percentage entering employment in Ireland, again an approximate number. This produced an FTE equivalent of graduates available for work in Ireland. This figure can also be corroborated with the register from the Irish Dental Council [31] as the register provides sufficient detail to establish the number of newly Irish trained and qualified dentists who have registered to practice in Ireland for the first time. Using the stock of dentists at the end of 1 year as a starting point for the next year, the numbers were adjusted for all inflows and outflows, as described above, to produce an estimate of the supply of dentists practising at the end of the current year. This became the starting stock figure at the beginning of the next year and the process of calculating inflows and outflows continued for each year thereafter of the planning period.

As stated previously, it is recognised that the Register of Dentists in Ireland includes dentists who may not be practicing. The September 2018 Register of Dentists includes 106 dentists (3.3% of total registered) who qualified before 1974 and are therefore assumed to be at least 65 years old. We have assumed that these dentists no longer practice. Additionally, there are dentists registered but who are in full-time academic positions and are not actively practicing. To account for this non-participation, we have assumed a ‘participation rate’ of 95%. Furthermore, with a growing trend towards improved work life balance among many dentists, there are increasing numbers of dentists choosing to work part-time hours. To account for this, and in the absence of verified statistics, we have applied an ‘activity rate’ of 85% (assuming 30% of dentists work part-time and work 50% of full-time hours). Provider supply figures, after incorporating all inflows and outflows, were adjusted accordingly.

To calculate dentist requirements for the population of interest, the Irish Eurobarometer 330 Oral Health dataset [33] was analysed in IBM SPSS Statistics 24 [34]. The sample data were analysed by gender, age cohort, health status and level of service, as described above, and then applied to both current population data and population projections published by and publicly available from the Central Statistics Office in Ireland [35]. The model simulated total provider requirements in minutes for 2017 and each year to 2050. From this, estimates of the population provider requirement (FTE equivalent) were produced for all years of the planning period assuming that practitioners spend 90% of their working hours providing direct patient care.

Having populated the model with all required data and run the simulation, we were then in a position to compare both present and future dentist supply and dentist requirements.

## Results

The number of dentists licenced to practice in Ireland in 2017, based on the Register of Dentists provided by the Irish Dental Association [31], was 3053. In the 2017 baseline scenario, the model calculated that 1985 FTE equivalents were providing general dental services to the adult population aged 15 years and over (provider supply). This figure excludes dentists working in the Public Dental Service, oral surgeons and orthodontists. It also accounts for annual flow, participation and activity rates as described above.

Using (i) population statistics for 2017 for persons aged 15 years and over [35], (ii) annual data on the number and types of dental visits from the Irish Eurobarometer 330 Oral Health dataset [29], provided in Table 2 below, and (ii) applying time per visit to each type of visit (assumed 20 min for a check-up/exam/cleaning, 30 min for routine treatment, 40 min for emergency treatment), the model estimates the total working hours required of dentists per annum. Assuming 1580 h are worked by dentists per annum (equivalent to 45 working weeks, 39 h worked per week and dentists spending 90% of their working time with patients), the model produces an FTE provider requirement figure of 899 dentists. Using an FTE provider supply figure of 1985, the model therefore suggests that provider supply is 2.2 times provider requirement. Using official Irish adult (15 years and over) population projections out to 2050 and keeping all baseline assumptions constant, the model projects the supply of primary care dentists to grow to 3987 by the year 2050 compared with a provider requirement figure of 1116.

**Table 2:**
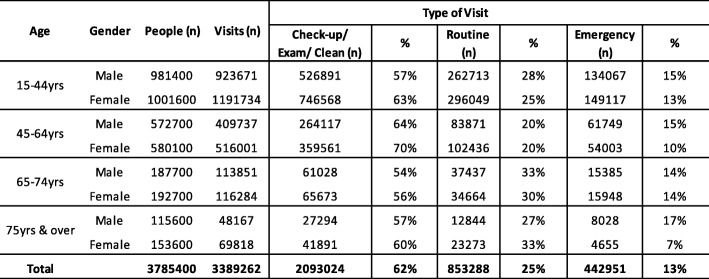
Type of dental visits by age cohort and gender in a 12-month period

**Fig. 2:**
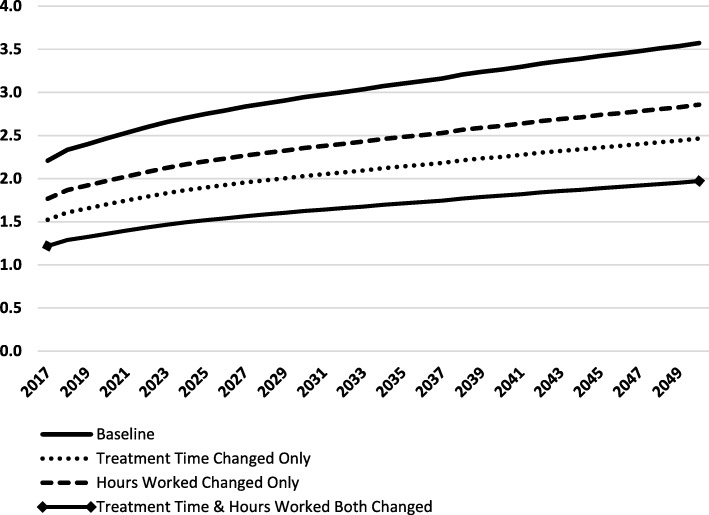
Model simulation output using Irish data: ratio of provider supply to provider requirement

As the output is hypothetical, based on many input assumptions required to activate the model, we conducted 3 alternative scenario analyses to demonstrate the impact of the assumptions on the model output. Firstly, in the absence of data on treatment time, the time per visit type was adjusted to 30 min for a check-up/exam/cleaning, 40 min for routine treatment and 60 min for emergency treatment. In this scenario provider requirement increases to 1303 FTEs. In the second scenario, it is recognised that parents may choose to pay privately for dental care for their children. As a result, many dentists in private practice have patients under the age of 15 years who have not been accounted for in the baseline scenario. To account for this, we assumed that a typical dentist spends 20% of their time with children. The hours worked per week have therefore been reduced to 80% of the baseline scenario (31.2 v 39 h) to exclude this care from the analysis. This increases the provider requirement to 1123 FTEs for serving the needs of the population aged 15 years and over. In scenario 3, we combined the adjusted times and reduced hours detailed above and the provider requirement increases to 1629 FTEs. Figure 2 illustrates the ratio of provider supply to provider requirement in each of these scenarios from 2017 up to the end of the planning period in 2050. These scenario analyses clearly demonstrate the significant impact that assumptions can have on workforce planning output when reliable data are not available.

## Discussion

In the context of the development of our needs-based oral health workforce planning model and the World Health Organization’s objective of aligning investment in human resources for health with the current and future needs of the population [36], a number of studies projecting health workforces which use demand-based and utilisation-based approaches are interesting to note. While one recent demand-based study conducted for OECD countries acknowledges that demand for health workers is influenced by changes in the epidemiologic conditions of a population, the empirical model put forward does not in fact include epidemiologic conditions at all [37, 38]. In a European context, it is reported that while Greece has the highest dentist to population ratio of EU countries, far above the EU average, oral healthcare remains expensive and unavailable to many citizens. Furthermore, the study highlights that simply using a dentist to population ratio as a measure to plan and allocate the dental workforce will result in oral health needs remaining unmet [39]. The situation in Greece highlights the complexities associated with the provision of integrated health services and workforce planning in trying to balance publicly and privately provided oral healthcare to ensure the oral health needs of the population are comprehensively served. A recent synthesis of analyses of workforce requirements in high-income OECD countries highlighted that there is evidence of inconsistent use of key workforce planning terminology, not least in terms of using ‘need’, ‘demand’ and ‘utilisation’ interchangeably which in turn affected the choice of method and quality of output in some studies [40]. Undoubtedly, there are challenges associated with planning human resources for health with differing schools of thought regarding the best approach to adopt. Efforts to date across the health sector have not demonstrated that they are fit for purpose or achieving the aim of ‘having the right people in the right place at the right time to treat the right people’ [1]. This failure to effectively develop and implement workforce planning across the health sector has associated risks which are not insignificant, including lives at risk, increases in morbidity, ineffective allocation of health service roles and inefficient allocation of public funds.

We believe that in the first instance, the workforce planning approach chosen must be consistent with the objectives of the health system. Therefore, where a health system’s objectives include addressing the healthcare needs of the population, then the workforce planning method chosen must incorporate population health measures and the potential for changes in these measures in order to adequately respond to these changes. However, current available evidence suggests that most European countries that do engage in model-based workforce planning do not take account of the health needs of the population [13, 40]. The model presented here therefore provides a starting point for the development of an oral health needs-based workforce planning tool.

It is recognised that the work that has been undertaken to develop and build this model is not without limitations. It is noted that many assumptions have been made to operationalise the model. Firstly, the model assumes that health status (number of natural teeth and problem with food or pain) by gender and age cohort will remain constant through the planning period, when in reality this will not be the case. While accepting that challenges faced with forecasting morbidity have necessitated this assumption, one might reasonably expect that this in fact results in an overestimation of provider requirement. For in planning the health workforce with an objective of meeting need, one would assume that health status will improve over time thereby reducing overall service use, in particular the need for time-consuming restorative and emergency dental treatment as opposed to less time-intensive preventive care, and as a result reducing overall provider requirement.

Secondly, we recognise that demand for private oral healthcare is evolving with an increase in demand for cosmetic services which lie outside healthcare needs. However, our model does not account for such services. As a result, additional workforce capacity will be required to meet these demands. This capacity can be estimated using traditional demand-based models, since these services are beyond meeting clinical need. The model presented in this paper is concerned with needs-based requirements for oral healthcare.

Thirdly, shortcomings have been identified around the availability of data that are required to operationalise such a model. Additionally, given the fact that dentistry is largely delivered by independent providers, there are challenges faced in obtaining more detailed information about the working practices of oral healthcare providers. However, in demonstrating the output possible with the limited public data currently available, our work also highlights the volume of data required to populate such a model, what data are currently publicly available and what data are lacking. The need for routine collection of both relevant oral health data in other contexts [41] and reliable data for workforce planning [16] has been highlighted previously. Until these data deficiencies and those identified through our work are addressed, it will impact on the ability of those charged with responsibility for workforce planning to successfully implement effective needs-based workforce planning for the oral health workforce. If future dental workforces are to contribute efficiently to population wellbeing, there is a pressing need for more comprehensive monitoring of the inputs, outputs and outcomes associated with the provision of dental care.

Lastly, in applying our model, we have assumed that all dental services provided to patients with primary care needs will be delivered by dentists only. We recognise that this may not reflect reality in all cases and acknowledge that there is increasing debate about the role of dental care professionals and the types of care they can effectively and efficiently deliver [42]. However, the model can be extended to allow for a different skill mix where some of these services may be provided by alternative providers, e.g. dental hygienists and dental therapists, thus reducing the requirements for dentists.

## Conclusions

There is ongoing recognition by policy makers of the pressing need to better plan human resources for health. In response to the scarcity of work done in this area specifically for dentistry and in line with our belief that one of the objectives of a health system must be to address the needs of its population, we have used an existing conceptual framework to develop a needs-based workforce planning simulation model for the oral health workforce. To demonstrate the workings of the model, we have applied the model in a hypothetical context using publicly available data where possible and have shown how the model compares provider supply to provider requirements to identify imbalances in the market for oral healthcare providers. We have also provided scenario analyses to demonstrate the impact that changes in the values of key inputs have on the model output. Although the results presented are hypothetical, most importantly we have demonstrated the feasibility of producing a useful, practical and workable oral health needs-based workforce planning simulation tool. The model has been developed with a focus mainly on public provision of dental care according to population oral health needs. For areas of dentistry showing recent increasing demand, such as cosmetic dentistry, which may or may not be considered for public provision of dental care in the future, the model can be extended accordingly to additionally incorporate different types of services. Additionally, the model is amenable to take account of technological advances in dentistry. Further development of the model will also allow for the addition of a variety of provider groups in a single setting, incorporating skill-mix changes and an analysis of the associated economic impact.

## Data Availability

The datasets analysed during the current study are available from the GESIS Leibniz Institute for the Social Sciences, at https://dbk.gesis.org/dbksearch/sdesc2.asp?no=4977 and the Central Statistics Office in Ireland at https://www.cso.ie/en/statistics/population/.

## Abbreviations

ADVOCATE: Added Value for Oral Care
FTE: Full-time equivalent
